# Potential Role of Exosomes in the Pathogenesis, Diagnosis, and Treatment of Ovarian Cancer

**DOI:** 10.3390/cancers18111690

**Published:** 2026-05-22

**Authors:** Anna Markowska, Michał Antoszczak, Janina Markowska, Adam Huczyński

**Affiliations:** 1 Department of Perinatology, Poznań University of Medical Sciences, Polna 33, 60-535 Poznan, Poland; 2 Department of Medical Chemistry, Faculty of Chemistry, Adam Mickiewicz University, Uniwersytetu Poznańskiego 8, 61-614 Poznan, Poland; 3 Gynecological Oncology Center, Poznańska 58A, 60-850 Poznan, Poland

**Keywords:** exosomes, ovarian cancer, cancer pathogenesis, in vitro, in vivo, liquid biomarkers, nanodelivery systems

## Abstract

Ovarian cancer (OC) remains one of the most lethal gynaecological malignancies, which is mainly due to late diagnosis, high frequency of metastasis, and the risk of developing resistance to systemic therapy. In recent years, exosomes—small extracellular vesicles (EVs) secreted by cancer cells and components of the tumour microenvironment (TME)—have been identified as potential mediators of OC progression. Exosomes participate in intercellular communication and enable the transfer of RNA, proteins, and lipids. These vesicles may modulate the immune response, promote angiogenesis, remodel the extracellular matrix, and drive epithelial–mesenchymal transitions. Exosomes also appear to play a role in the development of drug resistance via direct transfer of resistance factors or indirect modification of TME. In this review article, we summarise current knowledge on the biological role of exosomes in OC pathogenesis. We also discuss their possible diagnostic, prognostic, and therapeutic relevance. The properties and composition of exosomes make them promising noninvasive liquid biomarkers and convenient carriers for anticancer drugs. However, to fully exploit their potential, further large-scale preclinical and clinical studies are required, which should focus primarily on standardising research methods and assessing the safety and efficacy of exosome-based diagnostic and therapeutic methods.

## Simple Summary

Ovarian cancer (OC) is one of the deadliest cancers in women, primarily because it is often diagnosed at a late stage and can become resistant to treatment. Recent studies indicate that exosomes released, for example, by cancer cells, may play a significant role in the development and spread of OC. This review summarises current knowledge about the pathogenic role of exosomes and explores their potential use in OC diagnosis and treatment. Although exosomes could provide new options, further research is needed to verify their true potential in these areas.

## 1. Introduction

Ovarian cancer (OC) is a malignant gynaecological neoplasm with an unfavourable prognosis. According to the information from the international GLOBOCAN database, more than 320,000 women worldwide are diagnosed with OC each year [1], placing it among the ten most frequently diagnosed cancers in the female population [2]. At the same time, OC accounts for more than 206,000 deaths annually [1], representing nearly 5% of all cancer-related deaths in women [2]. In early-stage disease, the 5-year survival rate exceeds 90% [3]. Unfortunately, because the disease is asymptomatic in its early phase, up to 75% of cases are diagnosed at an advanced stage [4], resulting in a dramatic decrease in the 5-year survival rate to below 30% [5].

A characteristic feature of OC is its pattern of spread. Unlike most solid tumours, which metastasise mainly via the bloodstream or lymphatic circulation, OC may disseminate throughout the abdominal cavity by ascitic fluid [6]. Ascites constitutes a complex tumour-associated microenvironment in which dispersed cancer cells coexist with immune cells, mesothelial cells, and tumour-associated exosomes (TEXs) [7]. An additional clinical challenge is the marked heterogeneity of the disease [8], which hampers the development of universal and effective clinical strategies. Currently, OC treatment includes surgery, chemotherapy (first-line treatment based on platinum compounds and paclitaxel [9]), radiotherapy, and targeted therapies. Despite an often favourable initial response to treatment, most patients eventually experience disease recurrence, which in many cases leads to death.

One of the key challenges in OC treatment is also the lack of effective methods of early diagnosis. In clinical practice, transvaginal ultrasonography and CA125 serum concentration measurement are used [10]. However, this marker has limited specificity, as its elevated levels are also observed in non-neoplastic conditions, such as endometriosis [4]. A combined use of CA125 with the HE4 marker, expressed as the ROMA (Risk of Ovarian Malignancy Algorithm) index, increases diagnostic specificity [4], but it is still insufficient, particularly for the detection of early-stage malignancy. A randomised clinical trial showed that combined screening based on CA125 measurement and transvaginal ultrasonography may be insufficient, as it did not lead to reduced mortality compared with standard care [11].

**Figure 1:**
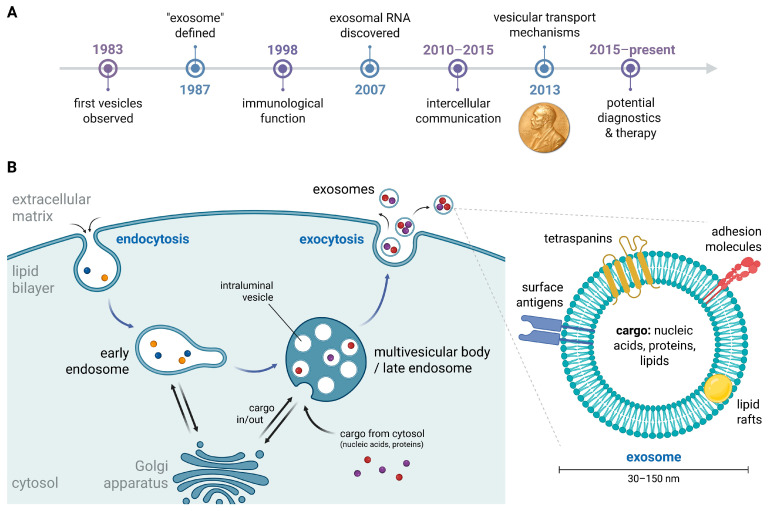
(**A**) History of exosome research, and (**B**) exosome biogenesis. Following endocytosis, internalised material is transported to the early endosome, which subsequently matures into the late endosome, also known as a multivesicular body (MVB). These compartments are distinguished by numerous intraluminal vesicles (ILVs) that arise through inward budding of the endosomal membrane. During this process, selected proteins, lipids, and cytosolic components are incorporated into these vesicles and may serve as future exosomal cargo. In addition, biomolecules can be delivered to MVBs from the trans-Golgi network and possibly from the cytosol. MVBs have two principal destinies: they either fuse with lysosomes, leading to degradation of their contents, or traffic to the plasma membrane. Upon fusion with the cell surface, the ILVs are released into the extracellular environment as exosomes. The figure was created with BioRender.com, accessed on 16 March 2026.

In recent years, increasing attention has been paid to the potential of exosomes for early OC detection [5,12,13,14]. Exosomes are a subpopulation of extracellular vesicles (EVs) first described in the early 1980s (Figure 1A) [15,16]. It has been shown that exosome isolation using anti-EpCAM or anti-CA125 antibodies yields a greater number of tumour marker-positive vesicles from oncology patients than from healthy individuals [17]. Chen et al. [18] have found that CA125 levels measured in the serum-derived exosomes are higher than serum CA125 levels. In turn, proteomic and lipidomic studies of exosomes derived from OC cells and normal ovarian epithelium, published in 2020, identified more than 1,400 proteins and more than 1,200 lipids, many of which differed significantly between the analysed cell lines [19].

Structurally, exosomes are nanovesicles of 30–150 nm in diameter (on average about 100 nm), surrounded by a lipid bilayer membrane (Figure 1B) [4,5,20,21]. Their primary function is to enable intercellular communication. They may be secreted by many cell types, including fibroblasts, stem cells, immune cells, and cancer cells [22,23]. Their release increases under pathological conditions [24,25], and it has therefore been postulated that they may participate in the transformation of normal cells into cancer cells and in the formation of pre-metastatic niches [26]. Exosomes are abundant in tumour tissue, the tumour microenvironment (TME), and numerous body fluids, including blood, urine, saliva, cerebrospinal fluid, ascites, tears, sweat, and breast milk [4,5,21].

The interior of exosomes is highly diverse and includes, among others, proteins associated with membrane transport (e.g., Rab GTPases, annexins), proteins involved in the formation of multivesicular bodies (MVBs), classical tetraspanins (CD9, CD63, CD81, CD82), adhesion proteins (CD106), and heat shock proteins (HSP60, HSP70, HSP90) (Figure 1B) [21]. Exosomes also contain nucleic acids such as microRNA (miR), circRNA, and long non-coding RNA (lncRNA), as well as sugars (Figure 1B) [21,27]. The exosome membrane and lumen are rich in lipids, including sphingomyelin, phosphatidylserine, phosphatidylinositol, and ceramide (Figure 1B) [21], playing an important role in their release. The composition of exosomes depends closely on the type of cells of their origin [28], which further contributes to their structural heterogeneity. Numerous methods are used to isolate and analyse these nanovesicles, including electron microscopy, Western blotting, immunoprecipitation of RNA-binding proteins, fluorescence in situ hybridisation, and specialised chromatographic techniques [12,14,29,30,31,32,33,34,35].

Exosomes exert pleiotropic effects by participating in intercellular communication, modulating cellular activity, and numerous signalling pathways [14,21,22,29]. They play an important role in immune responses, infections, inflammatory processes, neurological, metabolic, and cardiovascular diseases, and may also be involved in the progression of malignant tumours [14,21,22,29]. TEXs may interact with the cells present in TME, leading to their reprogramming and promoting their proliferation, invasiveness, and neovascularisation (angiogenesis) [36].

In OC, exosomes have been implicated in modulating multiple components of the TME, including alterations in macrophage function and other immune cell populations, thereby facilitating immune-stromal communication and promoting disease progression. The transfer of bioactive cargo, such as nucleic acids, lipids, and proteins, is the principal mechanism by which TEXs influence interactions both between cancer cells and between malignant cells and stromal compartments [5]. These processes are associated with several key biological outcomes, including (i) the acquisition of malignant traits by previously normal cells, (ii) attenuation of anti-tumour immune responses, (iii) stimulation of angiogenic pathways, and (iv) induction of epithelial–mesenchymal transition (EMT), ultimately enhancing metastatic potential [5].

In addition to their potential pathogenetic function, exosomes also exhibit considerable clinical potential. They may serve as noninvasive diagnostic and prognostic biomarkers in OC and, in the future, are expected to make specific and biocompatible drug carriers to be employed in a prospective targeted therapy strategy [14,21,22]. However, extensive (pre)clinical studies are needed to verify the potential of exosomes in this regard.

This review article summarises advances in understanding the relationship between OC and exosomes, based on scientific publications over the past 20 years, with particular emphasis on their roles in cancer progression, metastasis, modulation of TME, and mechanisms of drug resistance. The potential of exosomes as biomarkers in OC and as drug carriers in targeted therapy is also discussed. To this end, Google Scholar was searched in detail for original scientific papers using the keywords “ovarian cancer” and “exosomes”. The initial screening included articles containing the keywords in the title and/or abstract. Subsequently, all potentially relevant studies underwent full-text evaluation to determine their inclusion in the review. In addition, the bibliographies and content of the retrieved publications were analysed to identify previously overlooked studies relevant to this field. At this stage, we limited the inclusion criteria to exosome-focused and exosome-related studies.

The article therefore integrates current preclinical research data, drawing attention to the potential importance of exosomes in OC pathogenesis and their possible future application in personalised diagnosis and treatment.

## 2. Role of Exosomes in the Pathogenesis of Ovarian Cancer

The classification of EVs remains inherently complex and is still the subject of ongoing discussion within the field. EVs constitute a heterogeneous population that includes microvesicles, exosomes, and apoptotic bodies, which cannot always be unequivocally distinguished because isolation and characterisation methods are not universally standardised [37]. Consequently, although the International Society for Extracellular Vesicles (ISEV) recommends the collective term “extracellular vesicles” when vesicle subtypes cannot be precisely defined [38], many authors still refer to “exosomes” when focusing on vesicles enriched for endosomal origin markers and within the expected size range, particularly in tumour biology research. In this review, we use the term “exosomes” to remain consistent with the terminology used in the included original studies, particularly those reporting vesicles with exosome-like characteristics in OC.

In addition to their important role in normal body function, exosomes have also been implicated in the pathogenesis of OC. Tumour tissues are characterised by increased numbers of exosomes compared with normal tissues, which may show elevated interactions and intensive signal exchange between cancer cells and components of TME [39,40,41]. Exosomes derived from OC cells exhibited functional and molecular heterogeneity that led to differential biophysical and functional changes in epithelial OC-derived fibroblasts, including adhesion and migration, suggesting the development of a malignant TME [42]. In addition to their direct effects on cancer cells, the exosomes secreted by ovarian epithelial cells may also reprogram stromal cells by inducing the transformation of normal fibroblasts into cancer-associated fibroblasts (CAFs), among other mechanisms, through the transfer of miR-124 [43]. Reduced miR-124 levels promoted overexpression of the markers α-SMA and FAP, increased migration, and activation of SPHK1, whereas its overexpression reversed some of these changes [43]. Moreover, selected exosomal components (mainly non-coding RNAs and selected proteins) have been shown to stimulate uncontrolled cancer cell growth, modulate the immune response, promote angiogenesis, support metastasis from the primary site, and induce resistance to therapy (cytotoxic drugs) (Table 1) [14,21,22].

It should also be emphasised that not all exosomes exhibit the same biological activity. Studies by Chevillet et al. [89] have shown that exosome preparations contain only a small fraction of the total pool of microRNA present in the plasma of oncology patients. Moreover, most individual exosomes do not carry sufficient amounts of microRNA to induce a significant biological effect, suggesting that exosomes do not always act as independent and functional microRNA carriers [89]. On the other hand, the studies by Liang et al. [90] have evidenced that exosomal proteins derived from OC cells are overrepresented in signalling pathways associated with carcinogenesis, further indicating their importance in cancer progression.

The structure of this section reflects the major functional hallmarks of OC progression, including uncontrolled cancer cell growth, immune modulation, angiogenesis, metastasis, and therapy resistance. Although these processes are highly interconnected and may overlap in vivo, this framework enables a clearer presentation of the multifaceted roles of exosomes in OC.

### 2.1. Contribution to Uncontrolled Cancer Cell Growth

The exosomes derived from OC cells contain numerous bioactive molecules, including epigenetic regulators, specific proteins, and *N*-glycans, among which LGALS3BP has been identified [91]. They have also been shown to transport miR-205, which may increase the proliferation, migration, and invasion of target cells while inhibiting their apoptosis [61]. A similar effect was observed for exosomal miR-21-5p, which induced the same biological effects by regulating CDK6 at the mRNA and protein levels and, in in vivo models, led to increased tumour volume and mass [53].

Other RNA molecules transported in exosomes also play an important role in regulating OC cell growth. Exosomal lncRNA SOX2-OT was overexpressed in the plasma of patients with OC and promoted the proliferation, migration, and invasion of cancer cells while inhibiting apoptosis [48]. This mechanism operated through the SOX2-OT/miR-181b-5p/SCD1 axis, and its inhibition led to reduced tumour growth in an in vivo model [48].

Exosomes may also modulate TME. It has been documented that exosomes isolated from tumour-associated macrophages (TAMs) support the proliferation of OC cells while simultaneously inhibiting their programmed death [70]. MiR-589-3p in these exosomes affected OC progression by binding to BCL2L13, a protein that resembles the anti-apoptotic protein Bcl-2 [70]. Moreover, Li and Tang [64] have proved that the exosomes derived from M2 macrophages are rich in miR-221-3p, which inhibits CDKN1B expression, thereby supporting the proliferation of epithelial OC cells and the cell-cycle transition from the G1 to the S phase; low CDKN1B levels were associated with worse prognosis.

It should, of course, be borne in mind that not all biomolecules transported in exosomes enhance proliferation. Exosomal miR-940 secreted by OC cells acted as a suppressive factor, inhibiting cancer-cell proliferation, migration, and invasion and inducing apoptosis and G0/G1 cell-cycle arrest [92]. In addition, Amla (*Emblica officinalis*) extract has been shown to inhibit the invasive properties of OC cells by increasing miR-375 expression, which is also increased in the exosomes from cancer cells [93].

### 2.2. Contribution to Modulation of the Immune Response

TME is a dynamic multicellular system surrounding cancer cells and comprising diverse immune and stromal cells, vascular components, and extracellular matrix [94]. Within this microenvironment, exosomes play a key role in intercellular signalling, thereby significantly modulating the immune response and promoting cancer progression [94]. Heat shock proteins were elevated in exosomes from patients with gynaecologic malignancies and correlated with markers of cytotoxic immune response [95].

One important exosome population in TME is TEXs. After internalisation by immune cells, they may reduce immune activity through mechanisms including arginase-1-mediated suppression of T-cell proliferation or FasL-mediated induction of T-cell apoptosis [96,97]. In addition, CD47, identified as a tumour cell-derived exosomal signature, correlated with an immune-hot (inflamed) TME in OC, and was associated with activation of immune-related signalling pathways, and increased immune-cell infiltration [3]. At the same time, CD47 transfer via OC-cell exosomes has been shown to promote cancer cell escape from macrophage control and to support cancer progression [79]. Importantly, inhibition of exosome secretion or uptake increased phagocytosis by M1 macrophages and limited cancer dissemination within the peritoneal cavity [79].

The exosomes derived from OC cells may also directly affect the function of immune effector cells. They have been shown to suppress the immune response by reducing the expression of the NKG2D receptor on NK cells and peripheral blood mononuclear cells [86]. In addition, the OC exosomes recruited lymphocytes to the TME and modulated their functions, suppressing the anticancer response through IL10, Foxp3, and HLA-A/B, while simultaneously increasing the production of pro-inflammatory and pro-angiogenic cytokines such as IL6 and VEGFA [98]. These mechanisms involved activation of interferon pathways and NF-κB [98].

Modulation of macrophage function plays a particularly important role in forming the immunosuppressive TME. The exosomes containing the CMTM4 protein were internalised by macrophages, promoting their polarisation towards the immunosuppressive M2 phenotype [81]. This process led to activation of the NF-κB pathway, increased cytokine production, mainly TGF-β1 and CXCL12, and enhanced immune suppression in TME [81]. TEX-associated miR-222-3p, by reducing SOCS3 levels, also promoted macrophage polarisation towards the M2 phenotype, thereby creating an environment favourable for cancer progression [65]. The exosomes derived from TAMs contained microRNAs such as miR-29a-3p and miR-21-5p, which modulated the Treg/Th17 balance in CD4+ lymphocytes and, thus, induced an immunosuppressive microenvironment [54].

Hypoxic conditions may further enhance the immunomodulatory properties of OC exosomes. They were shown to deliver microRNAs that induce macrophage polarisation towards the M2 phenotype [99]. Hypoxia increased miR-940 expression in OC exosomes in both cell lines and patients’ ascites [71]. Exosomal miR-940, in turn, promoted macrophage polarisation towards M2, which subsequently supported cancer-cell proliferation and migration [71]. A similar effect was observed in the exosomes from the plasma of patients with OC, which showed elevated miR-200b levels that induced macrophage polarisation towards the M2 phenotype by suppressing KLF6 [60].

Ascites is also an important source of exosomes in OC. Peng et al. [100] have shown that exosomes were present in more than 85% of the patients examined and that they originated from various cellular sources. Although they did not directly affect cancer-cell proliferation or apoptosis, they significantly weakened the cytotoxic function of peripheral blood mononuclear cells in the presence of dendritic cells [100]. In addition, these exosomes induced apoptosis of both precursor and mature dendritic cells, as well as circulating immune cells [100].

Further studies indicate a key role of the ascites-derived exosomes in mechanisms of immune escape in OC. Shenoy et al. [74] have provided evidence that the exosomes present in ascites contributed to the formation of immunosuppressive TME, and identified ganglioside GD3 on their surface as a key factor that inhibits T-cell activation, irrespective of other exosomal components. Further studies have confirmed that the exosomes derived from the ascites of patients with OC may inhibit T-cell activation by blocking NF-κB and NFAT translocation and regulating activation marker expression, cytokine production, and cell proliferation; this effect was reversible and did not lead to the loss of cell viability [101].

At the molecular level, the exosomes derived from both cancer cells and immune cells may carry regulators of key signalling pathways. The TAMs-derived exosomes have been shown to carry the transcription factor GATA3, which modulates the immune response in the microenvironment of high-grade serous OC and promotes cancer progression [83]. In addition, exosomal miR-155-5p derived from OC cells was transported to macrophages and regulated PD-L1 expression, thereby affecting immune-cell migration and CD8+ lymphocyte activity [102]. Together, these findings are consistent with the evidence provided for OC, indicating that exosomes derived from TAMs contribute to immune evasion through the transfer of immunoregulatory molecules such as PD-L1, leading to suppression and exhaustion of T-cell activity [103]. Elevated levels of reactive oxygen species (ROS) in cancer cells reduced the miR-155-5p content of exosomes, which promoted the formation of an immunosuppressive microenvironment and cancer progression [102]. In OC models, targeting exosomal phosphatidylserine has been shown to reverse exosome-mediated immunosuppression, enhance CD4 and CD8 T-cell function, and reduce tumour burden and metastasis, highlighting a potential therapeutic strategy to restore anti-tumour immunity within TME [104].

Overall, TEXs are key mediators of immunosuppression and immune escape [105]; however, their elimination may enhance anti-tumour immunity. Notably, selected exosome populations may also stimulate cytotoxic T lymphocyte responses [106,107], highlighting their potential application in cancer immunotherapy, including exosome-based cancer vaccines and dendritic cell-mediated T-cell activation strategies [108].

### 2.3. Contribution to Angiogenesis

Angiogenesis is a key process underlying the growth and progression of malignant tumours [109], including OC. Exosomes may substantially contribute to angiogenesis through diverse molecular mechanisms, acting on both endothelial cells and components of TME.

For instance, in vitro and in vivo studies have shown that the HOXD11 protein present in these exosomes binds to the fibronectin (FN1) promoter, leading to increased VEGF expression and stimulating angiogenesis [84]. Analyses of exosomal molecular signatures also indicate an important role of lncRNA MALAT1 in supporting the expression of genes involved in angiogenesis in OC [47]. Exosomal MALAT1 expression was significantly higher in oncology patients than in the control group [47]. Exosomes transferred MALAT1 and promoted angiogenesis, with known pro-angiogenic factors such as VEGF-A, VEGF-D, IL-8, angiogenin, bFGF, and leptin acting as mediators of this effect [47].

The exosomes derived from highly malignant OC cells also stimulated endothelial cell proliferation, migration, and vascular structure creation, directly supporting angiogenesis [110]. Proteomic analysis of these exosomes identified proteins such as ATF2, MTA1, and ROCK1/2 that may be responsible for their angiogenic activity [110].

MicroRNAs transported in exosomes may also play an important role in regulating angiogenesis. He et al. [62] have shown that exosomal miR-205 actively modulated endothelial-cell functions, enhancing angiogenesis and promoting cancer progression via the PTEN-Akt signalling pathway. Similarly, the exosomes secreted by OC cells enhanced angiogenesis and endothelial-cell migration, and the microRNAs they contained, including miR-92b-3p, regulated this process by modulating SOX4 expression [56]. Exosomes also mediate paracrine communication between OC cells and endothelial cells, promoting angiogenesis by transferring miR-141-3p [59]. This microRNA reduced SOCS-5 expression, activated, among others, the JAK-STAT3 pathway, and increased VEGFR-2 levels, as a result promoting endothelial-cell migration and the formation of new blood vessels [59].

On the other hand, some exosomal microRNAs may exert anti-angiogenic effects. An example is exosomal miR-6126, which acted as a suppressor of cancer progression by regulating integrin β1, leading to reduced cell migration, invasion, and angiogenesis [111].

### 2.4. Contribution to Metastasis

OC metastasis is the main cause of failure of commonly used therapies and of the high mortality among patients [112]. Increasing evidence indicates that individual exosomal components may play a key role in regulating migration, invasiveness, and pre-metastatic niche formation in OC. For example, the integrin α3 presence has been documented in OC exosomes, and its intercellular transfer has been shown to increase metastatic potential in both OC cell lines (SKOV-3, OVCAR-3) and animal models [29]. Exosomal CMTM4 promoted macrophage M2 polarisation and NF-κB-mediated immunosuppression in OC, contributing to immune evasion and cancer progression, including metastasis [81].

Exchange of exosomes between cells with different invasive potential plays an important role in heterogeneous OC. Shen et al. [77] have indicated that the exosomes secreted by highly metastatic OC cells, containing the membrane protein CD44, increased the migration and invasiveness of cells with lower metastatic potential by transferring this protein and a metastatic phenotype. Moreover, the exosomes derived from OC cells transferred CD44 to peritoneal mesothelial cells, inducing phenotypic changes and increased MMP9 secretion, which promoted cancer invasion and dissemination [78]. Vesicles similar to exosomes secreted by OC cells also transported the soluble form of L1 (CD171), which activated the ERK pathway and could stimulate cancer-cell migration in both an autocrine and paracrine manner [80].

Exosomes may additionally enhance the aggressive features of OC cells by transferring microRNAs. The exosomes containing miR-328-3p were shown to increase the invasiveness of OC cells with lower metastatic potential by acting on Raf1 and disrupting the mTOR pathway [67]. Studies by Kobayashi et al. [113] further revealed that the SKOV-3 cell line, characterised by greater invasive potential, secreted nearly three times as many exosomes as the OVCAR-3 cell line. MicroRNA profile analysis demonstrated differences in the content of let-7 and miR-200 family transcripts in both the cells and their exosomes, suggesting that exosomal microRNAs may correlate with the invasive potential of cancer cells [113]. In addition, the exosomes derived from plasma cells transferred miR-330-3p to OC cells, inducing a change towards a mesenchymal phenotype by regulating JAM-B expression, which may promote aggressive cancer behaviour and tumour growth in vivo [68].

Exosomes may also play an important role in the modification of peritoneal mesothelial and stromal cells, thereby promoting metastatic implantation. Exosomal circPUM1 and circWHSC1 were transferred to peritoneal mesothelial cells, promoting OC metastasis and peritoneal dissemination, respectively [45,46]. In addition, the exosomes derived from OC cells transferred annexin A2 to mesothelial cells, inducing mesothelial-to-mesenchymal transition, extracellular-matrix degradation, and morphological changes and fibrosis via activation of the PI3K/Akt/mTOR pathway, consequently promoting the formation of a pre-metastatic microenvironment [75]. Exosomal piR-25783 derived from OC cells mediated communication with fibroblasts by activating the TGF-β/SMAD2/3 pathway and inducing their differentiation into myofibroblasts, contributing to fibroblast-to-myofibroblast transition and the formation of a premetastatic microenvironment in the omentum [73].

TEXs may transport miR-205 from OC cells to macrophages, consequently enhancing metastasis [63]. Moreover, TEXs increased miR-99a-5p levels, leading to elevated expression of fibronectin and vitronectin, thereby promoting cancer cell invasion and peritoneal dissemination in OC [58]. OC exosomes also carried lncRNAs that reversed the inhibitory effect of macrophage exosomes on endothelial-cell migration by modulating the miR-146b-5p/TRAF6/NF-κB/MMP2 axis [114].

Hypoxic conditions may further intensify the pro-metastatic properties of exosomes. The exosomes secreted by OC cells under hypoxia transported oncogenic proteins such as STAT3 and FAS, which increased cancer-cell migration and invasion, thus reprogramming fallopian-tube epithelial cells towards a protumorigenic phenotype [115]. In addition, exosomal LRG1, detected in the urine of patients with OC, promoted cancer-cell migration and disease progression by activating the FAK/Akt pathway, and its elevated level correlated with a worse prognosis [85].

One of the key mechanisms underlying metastasis is the EMT, the process in which cancer cells acquire mesenchymal features while losing epithelial characteristics [116]. EMT may promote the emergence of cancer stem cells (CSCs), which constitute 2–5% of the tumour-cell population and are characterised by self-renewal, migration, and resistance to treatment [117,118,119,120]. EMT is also associated with profound remodelling of the TME, based on complex interactions among proteins, microRNAs, lncRNAs, and other molecular factors [14]. Increasing evidence also indicates that TEXs may participate in specific processes supporting the microenvironmental changes required for EMT [121,122]. The exosomes isolated from the ascites of patients with OC could induce EMT through transfer of miR-6780b-5p [72], as could the ones derived from CAFs that contain TGF-β1, a key regulator in cell processes [88]. In addition, the exosomes secreted by OC cells with high LIN28A expression indirectly induced EMT in non-neoplastic cells, increasing their migration and invasion capacity [123].

### 2.5. Contribution to the Induction of Drug Resistance

Drug resistance is one of the main causes of therapeutic failure in OC treatment [124]. Increasing evidence indicates that exosomes may play an important role in this process, as they participate in the transport of diverse biomolecules between cancer cells and components of TME. The exosomes secreted by CP70 cells (a cisplatin-resistant OC cell line) were shown to induce increased resistance to this drug in the sensitive A2780 cell line [52]. It has been postulated that exosomes may act as mediators of OC-cell resistance to platinum drugs by transferring molecules that support cancer-cell survival and persistence [125].

TME plays an important role in modulating the response to chemotherapy [126]. Exosomal miR-21, isolated from cancer-associated adipocytes and CAFs, reduced the sensitivity of OC cells after transfer into these cells and inhibited apoptosis through interaction with the APAF1 protein [51]. The exosomes secreted by omental adipocytes also induced OC-cell proliferation and stimulated EMT, promoting an aggressive cancer phenotype [50]. The microRNAs they contained, including miR-21, let-7b, miR-16, and miR-92a, protected cells against paclitaxel, reducing their chemosensitivity [50]. Moreover, Zhu et al. [66] have proved that miR-223 transported by TAMs, particularly in hypoxic regions, promoted drug resistance in OC cells via the PTEN-PI3K/Akt pathway. Higher levels of this microRNA were also found in the serum of patients with the disease recurrence after taxol and cisplatin treatment, compared with the period after primary surgery, suggesting its association with OC recurrence [66].

Another mechanism of resistance transfer may involve exosomal microRNAs derived from CAFs. MiR-98-5p transported by CAF exosomes was shown to reduce CDKN1A expression in OC cells, increasing proliferation and inhibiting apoptosis, thereby promoting the development of cisplatin resistance both in vitro and in vivo [57]. Likewise, lncRNA UCA1 regulated cisplatin resistance through the miR-143/FOSL2 axis, and elevated UCA1 levels in the serum exosomes of treatment-resistant patients suggest a potential association with exosomes [49].

Studies of OC cell lines have identified additional microRNAs that may be associated with chemotherapy resistance. MiR-433 induced senescence in OC cells by downregulating CDK6 and reducing p-Rb levels, and could be released via exosomes to mediate a bystander senescence effect [69]. High miR-433 levels were associated with survival after paclitaxel treatment, indicating a role in chemoresistance [69].

In addition to RNA, exosomes may transport proteins that contribute to the induction of treatment resistance. Annexin A3, a protein linked to OC resistance to platinum-based therapy, may be secreted into exosomes [76]. The cells with high annexin A3 expression secreted increased numbers of exosomes, and the protein itself was detectable within them, highlighting a possible association of exosomes with resistance to platinum drugs [76]. In addition, cisplatin-resistant OC cells exhibited reduced lysosomal apparatuses and increased secretion of exosomes containing lysosomal proteins and drug transporters, such as MRP2, ATP7A, and ATP7B [127]. The exosomes from these cells accumulated considerably more cisplatin than those from sensitive cells, indicating an active mechanism of drug export from cancer cells [127].

The exosomes derived from OC cells also transported DNMT1, which, after transfer to other cells, promoted cisplatin resistance [82]. In addition, exosomal plasma gelsolin promoted OC-cell survival and cisplatin resistance through the autocrine and paracrine mechanisms [87]. In 2020, a significant role of circular RNAs was also demonstrated; exosomal circFoxp1 was elevated in the serum of patients with OC, especially in cisplatin-resistant cases, and correlated with clinical stage, metastasis status, and a worse prognosis [44]. Functionally, circFoxp1 promoted cancer-cell proliferation and induced drug resistance by modulating the miR-22/miR-150-3p axis and regulating CEBPG and FMNL3 expression, whereas its silencing increased cisplatin sensitivity both in vitro and in vivo [44].

An important component of the metabolic adaptation of OC cells to chemotherapy may be exosomal transfer of miR-21-5p [55]. It has been shown that miR-21-5p derived from cisplatin-resistant cells promoted glycolysis and reduced the chemosensitivity of sensitive cells by inhibiting PDHA1 expression [55]. Transfer of this microRNA by exosomes from SKOV-3/DDP cells increased cell survival and induced drug resistance, pointing to the potentially crucial role of the miR-21-5p/PDHA1 axis in OC pathogenesis [55].

## 3. Exosomes as Biomarkers

**Figure 2:**
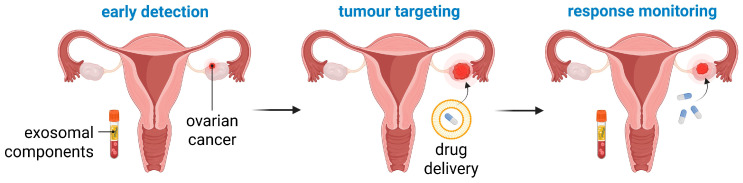
The multifaceted role of exosomes as potential liquid biopsy tools in ovarian cancer for detection, treatment, and monitoring. The figure was created with BioRender.com, accessed on 30 April 2026.

Beyond their biological role, an increasing number of studies indicate the diagnostic and prognostic potential of selected exosomal components, which can be isolated from both blood serum and other body fluids such as ascites or urine (Table 2) [13,14,21,128,129,130,131]. Exosomes display features specific to the cell type from which they originate and may therefore serve as potential disease biomarkers [132]. The multifaceted use of exosomes as biomarkers (Figure 2) has important advantages because they circulate in the body, which enables their collection in a minimally invasive manner [129]. In addition, they contain biomolecules that reflect the features of the parent cell and potential target cells, while simultaneously protecting the transferred informational biomolecules from degradation [129]. On the other hand, no single, specific biomarker for exosomes has yet been developed [24], although exosomes enriched in specific glycoproteins have been described in the literature and may serve as potential markers of these nanovesicles [133].

The best-characterised group of potential exosomal biomarkers in OC is microRNAs (Table 2). Analysis of exosomal microRNA may be a valuable diagnostic tool during the course of the disease [135,136,138,140,145,146]. The exosomes derived from OC effusions contain numerous microRNAs, some of which may have clinical significance and functional roles in disease progression, as they correlate with disease stage and patient survival [147]. Microarray analysis revealed a set of microRNAs differently expressed in both OC cells and their exosomes, with reduced miR-145-5p expression correlating with more advanced disease and a worse prognosis [148]. Among potential early plasma diagnostic markers of OC, the levels of exosome-contained microRNAs miR-21, miR-100, miR-200b, and miR-320 were significantly higher in patients with epithelial OC than in healthy women; additionally, miR-200b correlated with CA125 and patient survival and showed functional effects on cell proliferation and apoptosis [137]. Differences in the expression levels of selected microRNAs across studies (Table 2) [136,137,138] may, for example, result from differences in OC subtype, biological material (plasma versus serum), or patient cohort, illustrating the importance of method standardisation and cautious data interpretation.

Further studies identified miR-375 and miR-1307 present in the serum exosomes as potential diagnostic biomarkers of OC [141], as well as miR-373 and members of the miR-200 family (miR-200a, miR-200b, and miR-200c) [139]. Analysis of serum exosomes in 163 patients with OC showed that their concentrations were significantly higher than those in healthy women and were associated with disease stage and survival [139].

MiR-200 levels also enabled distinguishing cancers from benign tumours, and elevated concentrations of miR-200b and miR-200c correlated with lymph-node involvement, CA125 levels, and shorter overall survival, indicating their prognostic potential [139]. Yang et al. [149] have indicated that the serum exosomes from patients with OC contain miR-214-3p, whose level is higher in highly malignant neoplasms and in platinum-resistant tumors than in benign ovarian tumours. The miR-214-3p level correlated with the expression of target genes, such as LHX6, indicating the cancer’s biological features and showing its potential as a diagnostic and prognostic marker [149].

In addition to microRNAs, circular RNAs also show potential as biomarkers in OC. Profiling of circRNA in the serum exosomes from patients with OC showed significant dysregulation of their expression, with a clear increase in circ-0001068 expression compared with that in healthy individuals, and showed functional effects through exosome-mediated regulation of PD1 expression in T cells [134]. Circ-0001068 showed potential as a noninvasive diagnostic biomarker and was subsequently validated in a larger patient cohort [134]. In serous OC, an inverse relationship is also observed between the expressions of the cancer suppressor gene PDCD4 and the oncogenic miR-21, both in tumour tissues and in cells and exosomes from peritoneal effusions [150]. The presence of miR-21 in exosomes and its inverse correlation with PDCD4 may reflect cancer biology and indicate the potential significance of the PDCD4/miR-21 axis as a diagnostic biomarker in OC [150]. MiR-1290, in turn, showed significantly elevated expression in patients with high-grade serous OC compared with that in healthy individuals [142]. Its level was higher in advanced stages than in early stages (without statistical significance), and it decreased after surgery, suggesting that it may reflect tumour burden [142].

Exosomal protein biomarkers also appear to be an important area of research. The exosomes isolated from the blood of patients with OC showed a protein profile different from that of individuals without cancer (patients with pelvic floor dysfunction), and some of these proteins were also overexpressed in tumour tissue [151]. The exosomes containing the full-length claudin-family protein CLDN4 were detected in the plasma of patients with OC; at 98% specificity, the test’s sensitivity was 51% [144]. Other protein components of OC exosomes include, for example, EpCAM [152]. Nakamura et al. [153] showed that exosomal tetraspanins, particularly CD63, may correlate with disease stage, histological type, and response to chemotherapy, implying their potential for monitoring disease progression and treatment efficacy.

The exosomes containing CD147 (EMMPRIN) were detected in the ascites of patients with OC [154]; some studies, for example, in a colorectal cancer model, indicate that CD147 in circulating EVs can be directly detected in patient blood [155]. This creates prospects for their use as diagnostic markers and in translational research. At the same time, it has been shown that the level of CD24 in tumour tissue does not always reflect its content in exosomes [152], highlighting that exosomes may also constitute an independent source of biological information.

An interesting research direction is the quantitative and functional characterisation of exosomes. Lea et al. [143] have proved that the phosphatidylserine-exposing exosomes are significantly more numerous in the blood of patients with suspected OC than in healthy individuals and patients with benign tumours, and ROC analysis confirmed their high predictive value. In turn, Keserű et al. [156] have reported that the mitochondrial DNA copy number in the plasma exosomes from patients with OC is significantly increased in advanced-stage disease, indicating their potential as biomarkers of cancer progression.

## 4. Exosomes in Targeted Anticancer Therapy

Given that exosomes play an important role in OC progression, metastasis, and drug resistance, intensive studies worldwide have been focused on their reprogramming and use as endogenous drug carriers for targeted anticancer nanotherapy. Exosomes are promising candidates for non-toxic therapeutic carriers due to their relatively long circulation time, high biocompatibility, and ability to cross the blood–brain barrier [157]. In addition, exosome release by OC cells is regulated by a feedback mechanism [158], and modulation of this process may constitute a novel therapeutic strategy in OC treatment.

Exosomes may also serve as a platform for the development of innovative therapies, including strategies to improve the immune response in patients with advanced OC [159]. Literature describes an approach combining first-line chemotherapy with immunotherapy based on TLR3 receptor agonists and tumour-derived exosomes carrying tumour-associated antigens [160]. The exosomes derived from the ascites of patients with OC contained cancer antigens and cellular stress proteins that could be presented by dendritic cells derived from unrelated cord blood, leading to activation of resting T lymphocytes and induction of effective anticancer cytotoxicity [108].

Some exosomes may also exhibit direct suppressive properties against OC. For example, the exosomes rich in ADAM15 have been shown to inhibit cancer-cell migration and MEK/ERK pathway activation by proteolytically releasing the ADAM15 ectodomain, suggesting a potential role in regulating cancer progression [161]. Reza et al. [162] further showed that the exosomes derived from human adipose mesenchymal stem cell-conditioned medium inhibit the proliferation and migration of OC cells and induce apoptosis by modulating pro- and anti-apoptotic pathways. This effect was associated with the presence of exosomal microRNAs targeting molecules crucial for tumour survival [162].

An important research direction appears to be the use of exosomes as carriers of cytotoxic drugs. Doxorubicin-loaded exosomes increased the efficacy of OC treatment and reduced cardiotoxicity in mice [163]. The exosomes derived from mesenchymal stromal/stem cells and loaded with taxol showed strong in vitro cytotoxicity against the SKOV-3 cell line and significantly reduced tumour growth and metastasis in in vivo experiments despite the use of much lower drug doses [164]. Loading exosomes with the cancer-suppressive miR-199a-3p led to marked inhibition of proliferation, invasion, and tumour dissemination in experimental models [165].

The exosomes derived from immune cells seem to have particularly high therapeutic potential. Those from TWEAK-stimulated macrophages transferred miR-7 to OC cells, inhibiting the EGFR/Akt/ERK1/2 pathway and limiting metastasis both in vitro and in a mouse model [166]. The ones derived from M1 macrophages, especially those from cord blood, effectively delivered cisplatin to OC cells, increasing its cytotoxicity in both treatment-sensitive and treatment-resistant cells [167]. The exosomes derived from expanded NK cells, in turn, showed direct anticancer activity against OC, could serve as cisplatin carriers, and reversed NK-cell immunosuppression within the TME [168].

Despite promising results, the use of natural exosomes also has certain limitations (please refer to Section 5 for more information). Animal model studies have shown that intravenously administered exosomes have a short half-life of approximately 2 min [21], pointing to the need for their pharmacological modification. Exosomes may be administered directly into the tumour, intraperitoneally, or orally [21], but their pharmacokinetics remain incompletely understood. Exosomes may be engineered to enhance their targeting ability and therapeutic efficacy, for example, by loading them with bioactive molecules such as miRNAs, proteins, or drugs, or by modifying their surface to improve binding to specific cancer cells [169]. Such strategies enable the development of more efficient exosome-based platforms for targeted cancer therapy and immunomodulation.

In response to these limitations, biomimetic engineering strategies, among others, are being developed. For example, Pisano et al. [170] developed the immune-derived exosome mimetics that retain characteristic exosomal markers (CD63, CD81) and demonstrate higher efficacy in delivering doxorubicin to OC cells, while reducing drug dose and toxicity in 2D and 3D models. Li et al. [171] proposed hybrid nanoparticles based on tumour-derived exosomes that enable the co-delivery of miR-497 and triptolide to overcome cisplatin resistance. This system increased cancer-cell apoptosis, reduced toxicity, and exhibited strong anti-tumour activity in vivo, including inhibition of the PI3K/Akt/mTOR pathway, an increase in ROS production, and modulation of macrophage polarisation [171].

Additionally, the exosomes derived from umbilical-cord mesenchymal stem cells carrying miR-146a have been shown to increase the sensitivity of OC cells to docetaxel and taxanes and to inhibit proliferation in treatment-resistant models through the LAMC2-dependent PI3K/Akt pathway [172]. Exosomes have also been used as carriers of triptolide in OC models, demonstrating high encapsulation efficiency and stronger inhibition of proliferation in vitro and in vivo than the free drug, although liver and spleen toxicity was also observed [173].

## 5. Limitations and Future Perspectives

Although exosomes have emerged as important mediators of OC progression and as promising tools for diagnostic and therapeutic applications, their clinical translation remains constrained by several unresolved issues. A fundamental challenge is the intrinsic heterogeneity of EVs. Exosomes constitute only one subset within a broader vesicular spectrum that also includes microvesicles and apoptotic bodies [174]. In complex biological fluids, these populations often overlap in size, origin, and physicochemical properties, making precise discrimination difficult under current methodological constraints.

This problem is further compounded by the lack of universally accepted protocols for vesicle isolation and characterisation [27,175,176,177]. As a result, different experimental approaches often yield incomparable vesicle populations, contributing to variability in reported findings and limiting reproducibility across studies. In the context of OC, where biofluids contain mixed vesicular populations derived from multiple cellular sources, this issue is particularly pronounced.

Another important limitation concerns the highly diverse molecular composition of exosomes. Their cargo reflects a complex mixture of biomolecules, and their biological effects are unlikely to be driven by a single molecular species acting in isolation. Instead, their functional role appears to depend on the collective molecular signature of the vesicle population, which may vary according to cellular origin and microenvironmental conditions [178]. This perspective challenges the approaches focusing exclusively on individual components, such as single microRNAs, as standalone biomarkers or effectors. Detailed information on exosomal molecular cargo is available in dedicated exosome databases, such as ExoCarta (ExoCarta.org, accessed on 30 April 2026).

From a translational perspective, several technical barriers still limit their clinical application. These include limited availability of high-purity vesicle preparations, low natural abundance in biological samples, challenges in scalable production, and variable in vivo targeting efficiency [27,175,176,177]. Moreover, although engineered vesicles represent a promising avenue, most current strategies remain preclinical and require further validation before clinical implementation in OC patients.

Many published studies rely heavily on established cell line models and limited patient material, which may limit the robustness and broader applicability of the findings [24,37,157,179,180,181]. Consequently, further large-scale, well-designed clinical investigations are needed to strengthen current evidence. Important challenges also persist in exosome engineering, particularly in achieving precise and efficient incorporation of therapeutic agents. Existing loading approaches often suffer from low efficiency and limited reproducibility, and the functional consequences of modified exosomes in OC systems remain incompletely characterised [24,37,157,179,180,181]. Moreover, key aspects such as vesicle uptake mechanisms, structural stability, biosafety, and standardisation of isolation, preservation, and quality assessment remain insufficiently defined [24,37,157,179,180,181], all of which currently hinder clinical translation.

Despite these obstacles, the potential of exosomes in OC research remains substantial. They continue to be explored as minimally invasive biomarkers and as natural carriers for therapeutic delivery. Moving forward, progress in this field will depend on the development of more robust isolation techniques, improving the standardisation of analytical workflows, and implementing integrated, multi-level characterisation strategies. A more holistic understanding of vesicle biology, rather than a reductionist interpretation of individual molecular components, is likely essential for the successful clinical translation of exosome-based applications in OC.

At the time of analysis, several clinical trials (ClinicalTrials.gov, accessed on 30 April 2026) investigating exosome-based biomarkers and diagnostic or monitoring tools for OC were identified (Table 3). These studies predominantly originate from China and are largely in the early stages, with most trials still not recruiting or not yet posting results. Collectively, these data indicate that exosome-based clinical translation in OC is active but still emerging.

## 6. Conclusions

Exosomes are increasingly emerging as an important component of OC biology, playing a multidimensional role in intercellular communication and the dynamic regulation of the TME. Thanks to their ability to selectively transport microRNAs, lncRNAs, circRNAs, proteins, lipids, and other bioactive molecules, exosomes may enable signal transmission between cancer cells and components of the tumour stroma, including fibroblasts, adipocytes, immune cells, and endothelial cells. Consequently, they may influence modulation of the immune response, remodelling of the extracellular matrix, induction of angiogenesis, activation of EMT processes, and increased migratory and invasive capacity of cancer cells. These mechanisms may, in turn, promote disease progression, maintenance of the CSC phenotype, and the formation of metastatic niches within the peritoneal cavity and at distant sites.

The role of exosomes in the development of OC drug resistance appears to be particularly important. Transfer of exosomal microRNAs, drug-transporting proteins, metabolic enzymes, or signalling-pathway regulators may enable transmission of the resistant phenotype between cancer cells and adaptation of the tumour to therapeutic pressure. This phenomenon not only limits the efficacy of platinum-based chemotherapy but also promotes disease recurrence, which remains a major cause of therapeutic failure in patients with OC. Thus, exosomes may constitute an important element of the axes regulating treatment response and, at the same time, an attractive target for strategies to overcome drug resistance.

Moreover, numerous studies indicate that analysis of the molecular content of exosomes may provide valuable diagnostic and prognostic information. The exosomes isolated from blood, ascites, or urine exhibit characteristic profiles of microRNAs, circRNAs, and proteins that may correlate with disease stage, metastasis status, treatment response, and recurrence risk. Their presence in circulation and the possibility of obtaining them in a minimally invasive manner make them a promising source of liquid biomarkers, potentially enabling early detection of OC, patient stratification, and real-time monitoring of disease course. At the same time, the lack of well-defined markers specific exclusively to exosomes and the complexity of their cargo point to the need for further standardisation of isolation and analysis methods.

A further rapidly developing area of research is the use of exosomes as carriers of anticancer drugs and regulatory molecules. Their natural biocompatibility, ability to evade the body’s defence mechanisms, and potential for targeted delivery of therapeutic agents make exosomes an attractive alternative to synthetic nanocarriers. Preclinical studies suggest the potential to effectively deliver chemotherapeutics, cancer-suppressive microRNAs, or immunomodulatory molecules, thereby increasing treatment efficacy and reducing systemic toxicity. Nevertheless, the short circulation time of exosomes after intravenous administration, their incompletely understood pharmacokinetics, and the difficulties associated with large-scale production and modification remain major translational barriers.

In summary, exosomes may constitute an important and multifunctional component of OC pathogenesis, integrating the processes of cancer progression, drug resistance, and TME modulation. Their diagnostic, prognostic, and therapeutic potential makes them a novel and promising research target. However, the full exploitation of the opportunities offered by exosomes requires further extensive basic and clinical studies to better understand the mechanisms of their action and to develop standardised and safe clinical strategies. Appropriate control of exosome function may, in the future, enable the development of more precise, personalised diagnostic and treatment methods, thereby contributing to improved prognosis for patients with OC.

## Figures and Tables

**Table 1:** Potential pathogenic effects of selected exosomal components in ovarian cancer.

Biomolecule	Exosomal Cargo	Role in Pathogenesis	Ref.
circRNA	circFoxp1	Increasing cisplatin resistance	[44]
	circPUM1	Increasing metastatic potential	[45]
	circWHSC1	Promoting peritoneal dissemination	[46]
lncRNA	MALAT1	Supporting angiogenesis	[47]
	SOX2-OT	Enhancing cell migration, invasiveness, and proliferation, and inhibiting apoptosis, increasing tumour growth in vivo	[48]
	UCA1	Promoting cisplatin resistance	[49]
microRNA	let-7b	Reducing sensitivity to paclitaxel	[50]
	miR-16	Reducing sensitivity to paclitaxel	[50]
	miR-21	Reducing sensitivity to paclitaxel	[50,51]
	miR-21-3p	Supporting resistance to cisplatin	[52]
	miR-21-5p	Increasing cancer-cell proliferation, migration, and invasiveness; inhibiting apoptosis; increasing tumour volume, size, and weight in vivo; supporting immunosuppression; supporting resistance to cisplatin	[53,54,55]
	miR-29a-3p	Promoting immune escape; creating an immunosuppressive microenvironment	[54]
	miR-92a	Reducing sensitivity to paclitaxel	[50]
	miR-92b-3p	Modulating angiogenesis	[56]
	miR-98-5p	Promoting cisplatin drug resistance	[57]
	miR-99a-5p	Supporting cell invasiveness	[58]
	miR-141-3p	Promoting angiogenesis	[59]
	miR-200b	Promoting macrophage polarisation towards the M2 phenotype and inhibiting M1 polarisation	[60]
	miR-205	Increasing cell proliferation, migration, and invasiveness; inhibiting apoptosis; promoting angiogenesis	[61,62,63]
	miR-221-3p	Supporting cell proliferation	[64]
	miR-222-3p	Inducing macrophage polarisation towards the M2 phenotype	[65]
	miR-223	Increasing chemoresistance	[66]
	miR-328-3p	Increasing cell migration and invasiveness	[67]
	miR-330-3p	Increasing cellular aggressiveness and supporting cancer progression	[68]
	miR-433	Contributing to paclitaxel resistance	[69]
	miR-589-3p	Promoting cancer progression	[70]
	miR-940	Inducing macrophage polarisation towards the M2 phenotype	[71]
	miR-6780b-5p	Promoting EMT	[72]
piRNA	piR-25783	Promoting pre-metastatic niche formation	[73]
Lipids	ganglioside GD3	Promoting immunosuppression	[74]
Proteins	ANXA2	Promoting pre-metastatic niche formation	[75]
	ANXA3	Associated with resistance to platinum drugs	[76]
	CD44	Supporting cancer-cell migration and invasiveness	[77,78]
	CD47	Facilitating immune escape	[3,79]
	CD171	Supporting cancer-cell migration	[80]
	CMTM4	Supporting macrophage polarisation towards the M2 phenotype; enhancing secretion of immunosuppressive cytokines; promoting metastasis	[81]
	DNMT1	Increasing cisplatin resistance	[82]
	GATA3	Promoting proliferation and migration; supporting macrophage polarisation	[83]
	HOXD11	Promoting angiogenesis	[84]
	ITGA3	Increasing cell migration; supporting tumour growth	[29]
	LRG1	Associated with cell migration	[85]
	NKG2D ligands	Enabling immune escape	[86]
	pGSN	Increasing cisplatin resistance	[87]
	TGFβ1	Promoting EMT	[88]

**Table 2:** Role of selected exosome-associated components (cargo and surface molecules) as potential biomarkers in ovarian cancer.

Biomolecule	Biomarker	Material	Trend ^1^	Control Groups	Ref.
circRNA	circ-0001068	Serum	↑	Healthy individuals	[134]
microRNA	let-7a-5p	Serum	↑	Healthy individuals	[135]
	let-7b-5p	Serum	↑	Healthy individuals	[135]
	let-7c-5p	Serum	↑	Healthy individuals	[135]
	let-7d-5p	Plasma	↑	Healthy individuals	[136]
	let-7f-5p	Serum	↑	Healthy individuals	[135]
	miR-16	Plasma	↓	Healthy individuals	[137]
	miR-21	Plasma	↑	Healthy individuals	[137]
	miR-93	Serum	↑	Patients with benign and borderline ovarian masses	[138]
	miR-93	Plasma	↓	Healthy individuals	[137]
	miR-93-5p	Plasma	↑	Healthy individuals	[136]
	miR-99b-5p	Plasma	↓	Healthy individuals	[136]
	miR-100	Plasma	↑	Healthy individuals	[137]
	miR-106a-5p	Plasma	↑	Healthy individuals	[136]
	miR-122-5p	Plasma	↓	Healthy individuals	[136]
	miR-126	Plasma	↓	Healthy individuals	[137]
	miR-145	Serum	↑	Patients with benign and borderline ovarian masses	[138]
	miR-185-5p	Plasma	↓	Healthy individuals	[136]
	miR-200a	Serum	↑	Healthy women and patients with benign ovarian tumors	[139]
	miR-200b	Plasma	↑	Healthy individuals	[137]
	miR-200b	Serum	↑	Healthy women and patients with benign ovarian tumors	[139]
	miR-200c	Serum	↑	Patients with benign and borderline ovarian masses	[138]
	miR-200c	Serum	↑	Healthy women and patients with benign ovarian tumors	[139]
	miR-205	Plasma	↑	Healthy women and patients with benign ovarian tumors	[140]
	miR-223	Plasma	↓	Healthy individuals	[137]
	miR-320	Plasma	↑	Healthy individuals	[137]
	miR-342-3p	Serum	↓	Healthy individuals	[135]
	miR-373	Serum	↑	Healthy women and patients with benign ovarian tumors	[139]
	miR-375	Serum	↑	Healthy women and patients with benign ovarian tumors	[141]
	miR-574-3p	Serum	↑	Healthy individuals	[135]
	miR-877-5p	Serum	↑	Healthy individuals	[135]
	miR-1273f	Serum	↓	Healthy individuals	[135]
	miR-1290	Serum	↑	Healthy individuals	[142]
	miR-1307	Serum	↑	Healthy women and patients with benign ovarian tumors	[141]
	miR-4732-5p	Serum	↑	Healthy individuals	[135]
Lipids	Phosphatidylserine	Plasma	↑	Healthy individuals	[143]
Proteins	CLDN4	Plasma	+ ^2^	Healthy individuals	[144]

^1^ Trend (expression/biomarker level) in OC patients versus control group(s). ^2^ CLDN4 protein was present in 32/63 (51%) plasma samples from patients with OC versus 1/50 (2%) in healthy individuals.

**Table 3:** Clinical trials on the potential role of exosomes in ovarian cancer (ClinicalTrials.gov; online access on 30 April 2026).

Title	Location	Status	Results	No.
Exosome-based OCS scores for predicting ovarian cancer recurrence	China	Not yet recruiting	No results posted	NCT06558019
Exosome-based recurrence score for post-treatment ovarian cancer	China	Not yet recruiting	No results posted	NCT06925126
OCS products based on exosome technology were applied in the recurrence monitoring study after the initial treatment of baseline CA125-negative ovarian cancer	China	Not yet recruiting	No results posted	NCT07153705
Non-coding RNA in the exosome of the epithelial ovarian cancer	China	Unknown	No results posted	NCT03738319
A study evaluating the diagnostic performance of OCS in the differential diagnosis of endometriosis vs. endometriosis-associated ovarian cancer	China	Not yet recruiting	No results posted	NCT07029659
Combination therapy of senaparib and bevacizumab for first-line maintenance therapy in newly diagnosed advanced homologous recombination proficient ovarian cancer based on exosome protein marker	China	Not yet recruiting	No results posted	NCT07120451
Pilot study with the aim to quantify a stress protein in the blood and in the urine for the monitoring and early diagnosis of malignant solid tumors (EXODIAG)	France	Completed	No results posted	NCT02662621

## Data Availability

No data were used for the research described in the article.
